# MTF-NET: A mixed traffic flow multi-target detection network based on full-field perception and adaptive optimization

**DOI:** 10.1371/journal.pone.0344151

**Published:** 2026-03-16

**Authors:** Shihao Li, Qiao Meng, Xin Liu, Zhijie Wang, Siyuan Kong, Bingyu Li

**Affiliations:** 1 School of Computer Technology and Application, Qinghai University, Xining, Qinghai, China; 2 Intelligent Computing and Application Laboratory of Qinghai Province, Xining, Qinghai, China; Samsun University: Samsun Universitesi, TÜRKIYE

## Abstract

In mixed traffic flow scenarios, multiple types of traffic participants coexist on the same roadway, posing severe challenges for object detection algorithms due to significant disparities in target scales, complex background interference, dense occlusions, and the high heterogeneity of classes. Existing CNN-based detectors are constrained by the fixed receptive fields inherent in convolution operations and are generally plagued by imbalances between positive and negative samples as well as inadequate representations of small objects, further limiting their performance in mixed traffic detection tasks. To address these issues, we propose the MTF-NET detection network, which is endowed with full-field perceptual capabilities. First, a combination of CNN and MetaFormer is employed as the backbone for feature extraction to enhance contextual modeling. Second, to mitigate the inherent dual-dimensional information loss and small-target representation bottlenecks associated with pyramid structures, we introduce a Hierarchical Implicit-Explicit Pyramid structure alongside a Multi-Kernel Dilation Fusion Network designed to counteract the information degradation brought about by pooling operations. Finally, the Dynamic Dual Detection Heads utilize a dual-branch design that facilitates end-to-end deployment while alleviating the limitations imposed by non-maximum suppression (NMS), and a hybrid strategy integrating Exponential Adaptive Loss with Focaler-DIoU is developed to address the imbalance between positive and negative samples across multiple classes. Experimental results demonstrate that MTF-NET achieves a 5.1% improvement in mAP50 on the VisDrone2019 dataset, surpassing current state-of-the-art methods, and further yields enhancements of 4.2% and 13.4% on the UA-DETRAC-G2 and HazyDet datasets, respectively. These findings effectively validate the robustness and generalization capabilities of our network, providing a potent solution for object detection in complex mixed traffic flow scenarios.

## Introduction

With the rapid advancement of urbanization, mixed traffic flow [1] has emerged as a defining characteristic of modern urban transportation environments, encompassing a variety of participants such as pedestrians, bicycles, electric two-wheelers, and motor vehicles. The study of mixed traffic flow is critically important for urban planning, traffic management, intelligent transportation systems (ITS), and autonomous driving technologies. However, the significant heterogeneity in spatial distribution, dynamic characteristics, and speeds among different targets poses substantial challenges for object detection algorithms. In recent years, unmanned aerial vehicles (UAVs) have increasingly become indispensable for monitoring mixed traffic flows due to their high maneuverability, dynamic adaptability [2], and expansive field-of-view. Although existing detectors [3], such as Faster R-CNN, have made notable strides, their performance in detecting densely packed small targets from UAV perspectives remains unsatisfactory—especially when the per-frame target density exceeds 50, where severe occlusion and marked scale variations further complicate detection. Moreover, the high computational complexity of these algorithms constrains their deployment on embedded platforms. This disparity between algorithmic demands and hardware resources underscores the critical need for the development of lightweight yet robust detection frameworks. Consequently, the design of efficient and reliable object detection algorithms for mixed traffic flow holds significant practical importance for advancing intelligent transportation systems and enhancing UAV-based inspection efficiency.

Unlike region-proposal-based two-stage detectors, single-stage frameworks—such as the YOLO [4,5] series—have garnered significant attention in complex scenarios due to their efficiency. However, when addressing the multi-scale object detection task in mixed traffic flow scenarios, YOLO faces several key challenges: the conventional feature pyramid network (FPN) employs a top-down pathway along with lateral connections to fuse deep semantic information with shallow detailed features, yet the underutilization of high-resolution shallow features leads to a mismatch between semantic content and positional information for small objects. Furthermore, the severe loss of spatial details in deep features hampers the accurate localization of small targets in dense scenes, and the current models’ overemphasis on local receptive fields further diminishes detection accuracy. On the other hand, the reliance on non-maximum suppression (NMS) in traditional detection heads exhibits notable deficiencies; its greedy strategy may erroneously eliminate overlapping bounding boxes, resulting in missed detections of genuine targets. Although subsequent studies have introduced improvements such as Soft-NMS, their computational efficiency on embedded platforms remains suboptimal. These issues are particularly pronounced in mixed traffic flows characterized by dense interactions among pedestrians, non-motorized vehicles, and motor vehicles, thereby severely constraining the practical applicability of detection models.

In response to the aforementioned challenges, this study introduces MTF-NET, a multi-scale object detection network specifically tailored for mixed traffic flow scenarios. By synergistically integrating feature modeling enhancement, dynamic scale adaptation, and optimization mechanisms, MTF-NET overcomes the performance bottlenecks of existing methods in complex traffic environments. As demonstrated in [6], the spatial attention mechanism integrated in the YOLOv11n model exhibits significant advantages in recognizing lightly occluded targets and achieves outstanding performance in multi-class vehicle detection tasks [7]. Nevertheless, challenges such as small-object missed detections and localization bias persist in multi-class dense target scenarios. To address issues inherent in multi-scale feature modeling, this study proposes a feature enhancement network endowed with global perceptual capabilities. Specifically, we introduce ConvFuseFormer (CFF) and ContextAttentionFormer (CAF), which augment contextual information modeling by incorporating depthwise separable convolution (DCS) and vanilla self-attention mechanisms (VSA); replace the original module with a Multi-Kernel Dilated Fusion Network (MKDFN) that cascades dilated convolution layers configured with varying dilation rates to achieve continuous multi-scale receptive fields spanning from a local 3× 3 to an extensive 11× 11 range; and implement a dual detection head architecture that decouples positive sample point generation from the regression and classification processes, employing a learnable class-aware localization strategy that obviates the need for non-maximum suppression (NMS) post-processing. To tackle the challenge of small-object detection, we propose a novel Hierarchical Implicit-Explicit Pyramid (HIEP) structure, which establishes dual learning pathways for implicit feature enhancement and explicit feature reconstruction, and employs a progressive feature pyramid fusion strategy to recover local details under global semantic guidance. Furthermore, to mitigate the issue of imbalanced sample distribution in complex scenarios, we introduce the Exponential Adaptive Loss (EALoss) function, which constructs a dynamically weighted factor based on exponential decay to balance positive and negative samples, refines the evaluation metric into a Focaler Distance-IoU (Focaler-DIoU) function that incorporates a focusing mechanism, and achieves multi-scale adaptive localization through the integration of a scale-sensitive factor function. The primary contributions of this study can be summarized as follows:

1. In this paper, we employ a hierarchical embedding fusion strategy by synergistically integrating CNN with MetaFormer to construct a unified space-semantic modeling engine, which seamlessly coordinates local detail perception with global relational reasoning. This enables the development of a multi-target detection network backbone endowed with full-field-of-view capabilities, effectively addressing the challenge of drastic target scale variations in mixed traffic flow scenarios.
2. In this paper, by leveraging both spatial and frequency domain features to reconstruct small-target information, we introduce the HIEP structure along with a dual-path calibration mechanism. This approach enables the hierarchical fusion of multi-scale features and the effective restoration of small-target characteristics, thereby providing a robust solution to the inherent dual-dimensional information attenuation issue of pyramid architectures.
3. In this paper, by formulating a hybrid evaluation strategy that integrates the EALoss function with the Focaler-DIoU function, a dynamic sample modulation and multi-scale adaptive optimization mechanism is achieved, offering a novel approach to addressing the imbalance between positive and negative samples.

## Related works

### Feature extraction methods

Research efforts aimed at improving the YOLO architecture have predominantly focused on optimizing the network’s feature extraction structures. Meanwhile, with the evolution of CNNs, researchers have further integrated innovative designs into network architectures, such as the incorporation of depthwise separable convolutions [8], residual connections [4], and adaptive feature fusion techniques [9]. The trajectory of improvements rooted in traditional convolutional neural networks (CNNs) has become increasingly complex. Huixin Wu et al. [10] proposed the Coordinate Position Attention Module (CPAM) to enhance the effective utilization of features. Zhengxin Zhang et al. [11] realized effective fusion between low-level features and network features via depthwise separable convolutions, thereby boosting the network’s capacity for multi-scale feature learning. Ma, Chengji, et al. [12] adopted the Dense_CSPDarknet53 backbone, employing dense connections to extract latent image information; through structural re-parameterization and the ELAN strategy, they effectively mitigated background noise interference and improved feature extraction accuracy. However, the continuously increasing model complexity has significantly elevated deployment costs, rendering the construction of feature extraction architectures that balance accuracy and efficiency a key challenge in current research.

### MetaFormer and TransFormer

In recent years, Transformer [13] models have exhibited unprecedented potential across a variety of computer vision tasks [14,15], with their core capability widely attributed to the self-attention mechanism. Accordingly, numerous attention-based token mixers [16,17] have been proposed to enhance the performance of visual Transformers (ViTs) [15]. However, several studies [18,19] have demonstrated that substituting the attention modules within Transformers with simpler operators, such as spatial multilayer perceptrons (MLPs) [18,20] or Fourier transforms [19], can still yield satisfactory performance. Building upon this insight, the work [21] abstracts the Transformer as a lightweight and generalized architecture termed MetaFormer, hypothesizing that it is this MetaFormer framework that plays a critical role in high-performance models and thereby drives competitive outcomes. To validate this hypothesis, W. Yu et al. [21] employed an exceedingly simple operator—pooling—as the token mixer, observing that the resulting PoolFormer model substantially outperforms baseline models including ResNet, ViT, and MLP variants [15,18,20,22]. This finding further underscores the importance and superiority of the MetaFormer architecture. Moreover, Weiqi Yi et al. [23] applied the MetaFormer framework to decouple the token mixer and channel mixer in MobileNetV3, thereby further enhancing the model’s lightweight characteristics. To more effectively capture multiscale perceptual fields of diverse vehicles, Li Kang et al. [24] adopted mixed depthwise convolutions as the token mixer within MetaFormer (MDFormer). Collectively, these advancements substantiate the untapped potential of MetaFormer and Transformer architectures in the domain of object detection.

### Small target detection

Early approaches to small object detection primarily relied on handcrafted feature engineering and sliding window mechanisms. Models based on traditional feature descriptors such as HOG and SIFT exhibited fundamental detection capabilities in specific scenarios; however, they inherently suffered from limited cross-scene generalization. With the advent of deep learning, end-to-end detection frameworks exemplified by Faster R-CNN, YOLO, and SSD leveraged convolutional networks’ local invariance and multi-scale feature fusion mechanisms to achieve breakthrough performance in general scenarios. In recent years, researchers have continuously proposed innovative improvements tailored to diverse scenarios and requirements, thereby further expanding the applicability boundaries of small object detection methods. Dapeng Feng et al. [25] significantly enhanced the detection capability of small objects in aerial imagery by introducing a positional information encoding feature pyramid network (PieFPN), composed of complex and real encoders. Bhanbhro Hina et al. [26] proposed a multi-convolution block attention network (MCBAN) aimed at improving the detection accuracy of minute objects. Yi-Xin Huang et al. [27] presented the DQ-DETR model, which dynamically adjusts the number and spatial location of object queries by utilizing prediction maps and density maps from a classification counting module. Furthermore, Xue Chen et al. [28] introduced the sparse connected asymptotic feature pyramid network (SCAFPN), which enhances small object detection precision while ensuring model lightweightness. Nonetheless, in complex traffic scenes, existing methods still struggle to effectively detect small targets with scales below 16× 16 pixels, a fundamental challenge arising from the mismatch between the spatial resolution limitations of feature extraction networks and the continuous scale variations of targets in dynamic environments.

## Methods

### Overall framework

**Fig 1:**
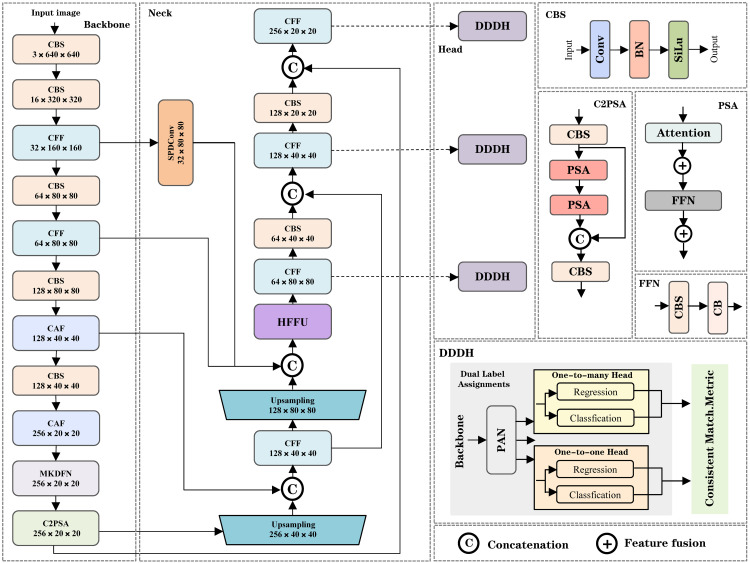
Overall Architecture and Inference Pipeline of the MTF-NET Detection Framework.

As illustrated in Fig 1, the proposed MTF-NET framework is built upon the YOLOv11n baseline, specifically engineered to address the challenges of multi-scale object detection in mixed-traffic scenarios. The architecture processes the input image through a hierarchical workflow comprising three synergistic components: a feature modeling backbone, a feature fusion neck, and a dual-branch detection head.

**Feature Modeling and Enhancement (Backbone).** The feature extraction stage (Backbone) transforms the input image (default 640×640 ) into multi-scale feature maps. To overcome the limitations of standard convolutions in capturing long-range dependencies, we integrate the Collaborative Architecture of CAF and CFF modules within the backbone. Following the MetaFormer paradigm, these modules deeply fuse global semantic information from Transformers with local spatial details from grouped convolutions. This design facilitates robust feature interaction across diverse spatial modalities, enhancing the correlation between fine-grained local features and macroscopic semantics. Furthermore, at the deep stages of the backbone, we introduce the MKDFN. By leveraging shared convolution kernels with dynamic dilation rates, MKDFN adaptively balances feature fidelity and computational efficiency, thereby optimizing texture feature extraction for objects of varying scales.

**Hierarchical Feature Fusion (Neck).** To address the representation degradation of small objects during feature fusion, the Neck employs a HIEP structure (manifested as the specific arrangement of CFF, CBS, and upsampling modules in Fig 1). The HIEP establishes cross-layer interaction channels via a dynamic weighting mechanism, which effectively suppresses information loss during downsampling and ensures semantic consistency between high-resolution (80×80 ) and low-resolution (20×20 ) layers. This structure allows for the precise reconstruction of spatial details crucial for detecting small-scale traffic participants.

**Dual-Head Inference and Optimization.** Finally, the processed features are fed into the Dual Detection Head (DDDH). Unlike traditional YOLO architectures that rely heavily on NMS, our design incorporates a dual-head mechanism: a One-to-many head for rich supervisory signals during training and a One-to-one head for end-to-end inference. This eliminates the latency overhead associated with NMS. The training process is governed by an improved hybrid loss framework, which combines an Exponential Moving Average (EMA)-based adaptive weighting mechanism with an enhanced DIoU metric, dynamically balancing class samples while reinforcing bounding box center constraints.

### Full-field perception feature enhancement network backbone

#### The architectures of ContextAttentionFormer and ConvFuseFormer.

**Fig 2:**
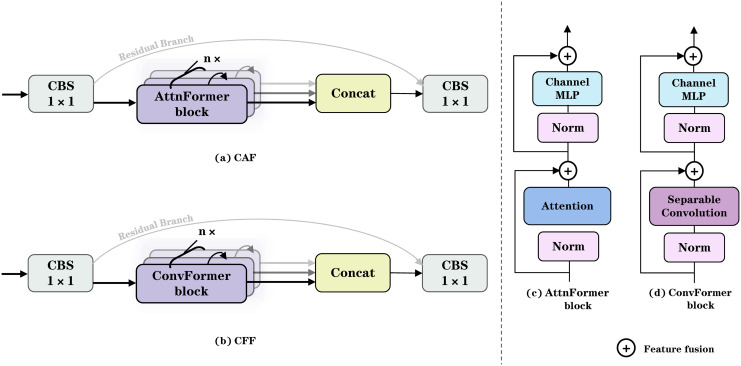
Overall architecture diagrams of CAF and CFF.

**Fig 3:**
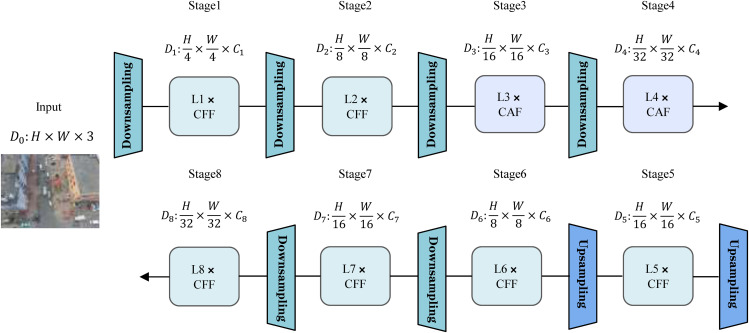
Overall architectural diagram of CFF and CAF in MTF-NET.

This paper embarks on an innovative exploration of the key challenges in deep network architecture design since YOLOv3 [4]. Although the evolution of the YOLO series has benefited from residual structures that effectively mitigate the vanishing gradient problem through connectivity—and with mainstream CNN architectures such as ResNet [22] and FasterNet [29] deepening networks to expand the receptive field and enhance global feature representation—the residual connection mechanism within the C3K2 module exhibits a bias in feature transmission. Specifically, the dominant propagation of high-level semantic features suppresses the retention of shallow, high-resolution spatial details, leading to a significant loss of fine-grained spatial information that is critical for detecting small objects (smaller than 20× 20 pixels). To address this issue, we propose two novel architectures, CFF and CAF, as replacements for the original C3K2 module (as illustrated in Fig 2), thereby achieving a systematic enhancement in multi-scale detection performance through a hierarchical feature augmentation strategy, with the overall network backbone depicted in Fig 3. In the shallow stages of the backbone, we introduce a CFF module integrated with DCS [30]. This module employs an efficient combination of grouped and pointwise convolutions to finely extract local spatial features while maintaining parameter efficiency, and its enhanced local receptive field properties significantly improve the discrimination of fine details such as object edges and textures.

Specifically, given an input feature map $X\in {\mathbb{R}}^{B\times C_{1}\times H\times W}\;$, where *B* denotes the batch size, $C_{1}\;$ represents the number of input channels, and *H* and *W* correspond to the height and width of the feature map, respectively. Initially, a 1× 1 convolution is applied to perform channel expansion, yielding feature $Z=f_{cv1}(Xin{\mathbb{R}}^{B\times 2c\times H\times W}\;$. Subsequently, $Z=f_{cv1}(Xin{\mathbb{R}}^{B\times 2c\times H\times W}\;$ undergoes channel splitting to produce $y_{0},y_{1}=Split(Z$. Thereafter, the features are subjected to a hierarchical nested structure for multi-level enhancement, followed by a final 1× 1 convolution to conduct channel reduction, as shown in Eq (1).


$$X_{out}=f_{cv2}\left(Concat\left(y_{0},\;y_{1},\;T_{1},\;\ldots ,\;T_{n}\right)\right)\;$$ (1)

To address the requirement for deep-layer global feature modeling within the backbone network, we innovatively design the CTF module integrated with VSA [13]. By employing the Query-Key-Value mapping mechanism, it constructs a semantic correlation graph across spatially disparate pixels, wherein the attention weight matrix simultaneously captures long-range semantic dependencies and spatial relationships. This capability enhances the model’s robustness to occluded objects and complex backgrounds, thereby further improving both the recall rate and localization accuracy in object detection. Given the input feature map X, it is first permuted to obtain $x^{\prime }=Permute(Xin{\mathbb{R}}^{B\times H\times W\times C}\;$. Subsequently, after applying layer normalization, a Token Mixer operation $t=\text{TokenMixer}(LN_{1}(x^{\prime })$ is performed. Here, $LN_{1}\;$ denotes a layer normalization operation designed to enhance model stability and accelerate convergence. The Token Mixer leverages a Query-Key-Value mapping mechanism to generate a feature representing the semantic correlations across spatially distant pixels. The output t, resulting from the Token Mixer, effectively captures the semantic relationships among the input features. A residual connection $r_{1}=x^{\prime }+\alpha_{1}\cdot D(t$ is then employed to strengthen feature representation. Within this context, D(t signifies the feature signal processed through normalization and the attention mechanism, while $\alpha_{1}\;$ represents a learnable scaling factor used to modulate the influence of the Token Mixer’s output on the overall feature. This residual connection scheme enables the model to integrate additional information obtained via the Token Mixer while preserving the original features, thereby enhancing the model’s representational capacity. Subsequently, after a second layer normalization, a multilayer perceptron (MLP) $m=MLP(LN_{2}(r_{1})$ is applied, where $LN_{2}\;$ serves to further normalize the features. The MLP comprises fully connected layers and activation functions, designed to perform deeper nonlinear transformations on the input features. The output *m* constitutes a refined representation of the preceding features, capturing more complex feature abstractions.Finally, through another residual connection and permutation operation, the final output feature $x_{out}={Permute}^{-1}(r_{2}$ is obtained. Moreover, to systematically address the contradiction between local and global features in mixed traffic flow detection, this work further proposes a dual-path enhanced architecture, as illustrated in Fig 2. The architecture deploys the enhanced modules separately along the downsampling and upsampling paths: within the downsampling path, a combination of two CCF modules and two CTF modules is employed to facilitate the coordinated extraction of local details and global semantics; conversely, four CCF modules are arranged along the upsampling path to emphasize multi-level enhancement of fine-grained edge information. This dual-path collaborative design not only achieves dynamic fusion of multi-scale features but also effectively mitigates the representational conflict between shallow spatial details and deep semantic features through a cross-path attention calibration mechanism.

#### Architecture of the Multi-Kernel dilation fusion network.

**Fig 4:**
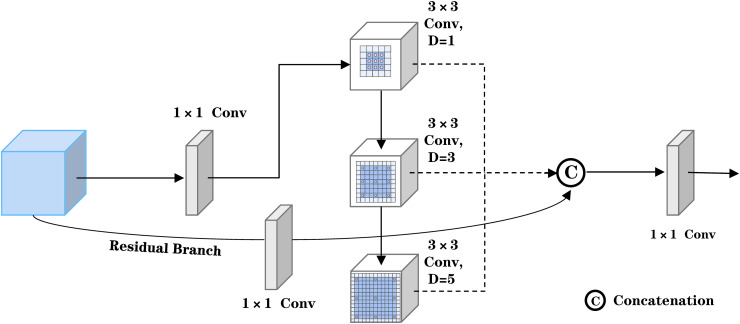
Detailed Architecture Diagram of MKDFN.

We removed the original SPPF module, which utilizes cascaded pooling kernels of varying scales to implement multi-scale max pooling. While this mechanism effectively reduces the spatial dimensions of feature maps via local maximum value selection and thus improves computational efficiency, max pooling retains only local extrema, resulting in attenuated fine structural details. Furthermore, the independent pooling operations do not fully capture the correlations among features across scales, leading to computational redundancy. Particularly in small object detection scenarios, the spatial resolution reduction inherent in pooling operations blurs the representation of small objects, and the suppressive effect of max pooling on fine-grained features makes object boundaries susceptible to being obscured by background noise or large-scale object features. To address these issues, we propose the MKDFN as a replacement for the original SPPF structure. The detailed architecture of the MKDFN module is illustrated in Fig 4.

The design employs a cascaded configuration of multiple dilated convolutional layers with progressively increasing dilation rates, incrementally elevating the dilation parameter at each stage to expand the receptive field while preserving spatial resolution. Given an input feature map *x*, it first passes through a 1× 1 convolutional layer cv1, yielding a latent feature map $y_{0}=cv1(xin{\mathbb{R}}^{B\times c_{x}\times H\times W}\;$. Subsequently, a series of dilated convolution operations are performed by iteratively traversing multiple dilation rates d∈dilations , as formulated in Eq (2).


$$\begin{array}{cc} y_{i}=\; & F.conv2d(y_{i-1},\;weight={share}_{conv.weight},\\ & \;bias=None,\;dilation=d,\\ & \;padding=\left(\frac{d\cdot (3-1)+1}{2}\right))\; \end{array}$$ (2)

This cascaded architecture enables the model to capture multi-scale feature representations at each layer, ranging from local textures to global context, thereby fully leveraging the advantages of dilated convolutions. Consequently, the output feature map $y_{output}\;$ is obtained as expressed in Eq (3).


$$y_{output}=cv2(Concat(y_{0},\;y_{1},\;\ldots ,\;y_{n}))\in {\mathbb{R}}^{B\times c_{2}\times H\times W}\;$$ (3)

Compared to conventional pooling-based downsampling strategies, the proposed method effectively preserves high-frequency details by circumventing spatial dimension reduction. The utilization of shared convolutional kernel parameters facilitates an optimized allocation of computational resources, enabling the model to simultaneously capture features at multiple hierarchical levels, thereby significantly enhancing the integrity of feature representation.

#### Dynamic dual detection heads structure.

In addition, this study addresses the dual challenges of computational efficiency and detection accuracy associated with the NMS algorithm in traditional detection frameworks. The conventional NMS algorithm exhibits an O(N²) time complexity, and in mixed traffic scenarios, the exponential increase in candidate boxes due to densely distributed targets makes it infeasible for embedded or edge computing platforms to meet real-time detection demands. Moreover, NMS is prone to erroneously discarding detection boxes when targets overlap. To resolve these issues, we adopt the detection head architecture proposed by Wang, Ao, et al. [31], herein referred to as DDDH. This architecture preserves the diversified supervisory capability of the original one-to-many branch to enhance feature generalization, while introducing a one-to-one detection branch to improve localization accuracy through precise label matching. During training, the one-to-many branch reinforces feature learning via multi-scale supervision, whereas the one-to-one branch optimizes target localization through bipartite graph matching based on the Hungarian algorithm. At inference, only the optimized single branch is activated, thereby maintaining detection precision and eliminating the computational overhead of NMS. The DDDH facilitates end-to-end integration of feature decoding and detection output, obviating the traditional NMS post-processing step and effectively mitigating the risk of missed detections due to bounding box suppression.

### Hierarchical implicit-explicit pyramid architecture

**Fig 5:**
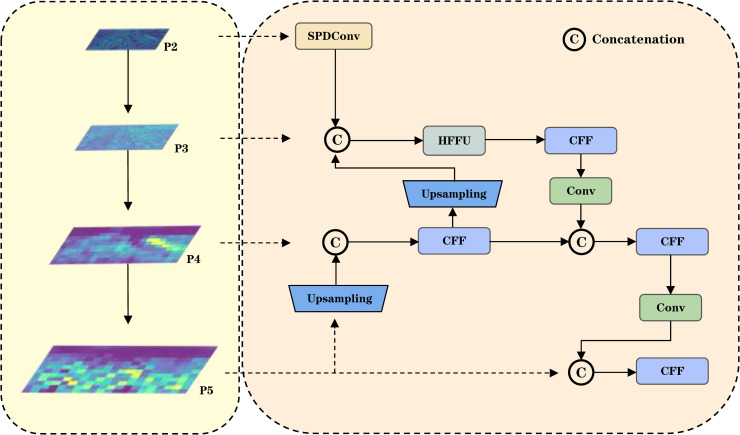
Overall structure diagram of HIEP.

**Fig 6:**
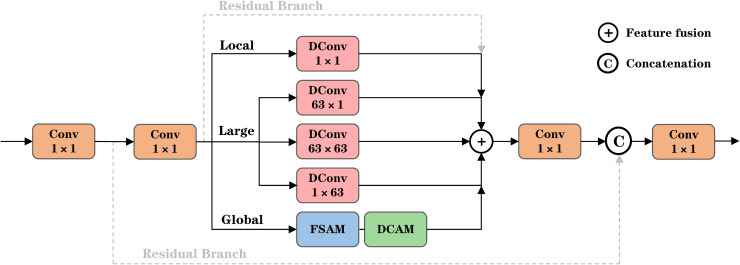
Internal Structure Diagram of HFFU.

In densely populated multi-scale target scenarios typical of mixed traffic environments, feature pyramids often encounter the issue of scale ambiguity, where deep features, dominated by large objects, tend to suppress the activation responses of small targets. Grounded in the information bottleneck theory [32], we identify a dual-dimensional attenuation phenomenon inherent to feature pyramid networks: spatially, progressive downsampling leads to the degradation of geometric information; channel-wise, feature compression results in weakened semantic representations. To address the geometric detail loss encountered during high-level feature fusion in conventional FPNs [33], we propose a HIEP tailored for small object detection, whose architecture is illustrated in Fig 5. This design synergizes an indirect high-level feature fusion mechanism with a multi-granularity feature integration framework, leveraging information exchange across implicit hierarchies alongside explicit multi-scale feature alignment. Consequently, it substantially enhances the geometric detail and semantic representation of small objects without necessitating additional detection layers. Concurrently, based on OmniKernel [34], we redesigned a Heterogeneous Feature Fusion Unit (HFFU) for multi-granularity feature integration, the detailed architecture of which is illustrated in Fig 6.

During processing, the input feature $X\in {\mathbb{R}}^{B\times C\times H\times W}\;$ is partitioned into two parts, $ok_{branch}\;$ and *identity*, via the convolutional layer cv1, as formulated in Eq (4).


$$[\begin{array}{c} ok_{branch}\\ identity \end{array}]=torch.split\left(cv1(X),\;\left[\left\lfloor C\cdot e\right\rfloor ,\;\left\lceil C\cdot (1-e)\right\rceil \right],\;dim=1\right)\;$$ (4)

Here, *e* serves as a hyperparameter, typically set to 0.25. Subsequently, the partitioned feature $ok_{branch}\;$ is fed into HFFU for further processing. Within HFFU, the input feature map $ok_{branch}\;$ is first processed by an input convolutional layer to produce $O_{in}=GELU(in_{conv}(ok_{branch}))\;$. Thereafter, the feature map undergoes a two-dimensional Fourier transform, as expressed in Eq (5).


$$O_{fft}=ℱ\left\{O_{in}\right\}=torch.fft.fft2\left(O_{in},\;norm=\text{\textbackslash{}textquotesingle backward\textbackslash{}textquotesingle}\right)\;$$ (5)

To enhance channel-wise features, the channel attention $x_{att}={fac}_{conv}({fac}_{pool}(O_{in})$ is computed and subsequently applied to the frequency-domain feature $O_{fft_{att}}=x_{att}\cdot O_{fft}\;$. An inverse Fourier transform is then performed to revert back to the spatial domain, yielding the feature weighted by channel attention, as delineated in Eq (6).


$$O_{fra}={ℱ}^{-1}\left\{O_{fft_{att}}\right\}=torch.fft.ifft2(O_{fft_{att}},\;dim=(-2,\;-1),\;norm=\text{'backward'})\;$$ (6)

Ultimately, the feature $|O_{fca}|\;$ is obtained. Subsequently, through the processing of the spatial-channel attention (SCA), features $x_{\text{attsca}}=\text{conv}\;\left(\text{pool}\left(O_{\mathrm{fca}}\right)\right)$ and $O_{sca}=x_{{att}_{sca}}\;\cdot O_{fca}\;$ are computed. The output Osca  is then processed by the FGM module to produce $O_{final}=FGM(O_{sca}$. Finally, during the feature fusion stage, the original input features are combined with the outputs of multiple deep convolutional layers, as explicitly formulated in Eq (7).


$$O_{out}=O_{in}+\sum_{i=1}^{5}{dw}_{i}\left(O_{in}\right)+O_{final}\;$$ (7)

Here, $dw_{i}\;$ denotes distinct depthwise convolution operations. Following the application of the ReLU activation function, the final output is obtained as specified in Eq (8).


$$Y={out}_{conv}\left(ReLU\left(O_{out}\right)\right)\;$$ (8)

To address the issue of information degradation within the feature pyramid, we further propose a dual-path calibration mechanism. This mechanism compresses the high-resolution features of the P2 layer via SPDConv [35] and subsequently transmits them to P3, thereby preserving edge and corner information of small objects. Concurrently, the upsampling process of deep features provides category-aware priors that suppress background noise interference, enabling top-down semantic guidance.

### Construction of an adaptive loss function

#### Exponential adaptive loss function.

In conventional object detection frameworks, the utilization of fixed-weight loss functions proves inadequate for effectively mitigating the imbalance between positive and negative sample distributions in mixed traffic flow scenarios. In the context of multi-scale object detection tasks, the original loss function employs fixed weighting coefficients that cannot adaptively adjust the contributions of individual loss components; however, targets at different scales exhibit varying sensitivities to localization and classification losses during optimization. Simultaneously, small object detection demands higher localization accuracy, as positional deviations may lead to detection failures; however, the fixed-weight strategy is incapable of dynamically modulating the contribution of loss terms according to object scale, thereby impeding the model’s ability to achieve balanced performance across different scales. This limitation motivates the adoption of the SlidLoss function, originally proposed by Yu, Ziping, et al. [36] in the domain of face recognition, as formally defined in Eq (9).


$$\begin{array}{c} f(x)=\left\{ \begin{array}{cc} 1, & x\leq \mu -0.1\\ e^{1-\mu }, & \mu -0.1<x<\mu \\ e^{1-x}, & x\geq \mu \end{array}\right.\; \end{array}$$ (9)

Here, *x* denotes the Intersection over Union (IoU) between the predicted bounding box and the ground truth, while μ represents the weighting threshold. In this paper, we propose the EALoss function based on the EMA theory [37], which effectively mitigates critical shortcomings of the original YOLO loss function in real-time detection under mixed traffic flow conditions. By leveraging a dynamic weight adjustment mechanism, the model progressively enhances its capacity to learn from hard samples during training, thereby overcoming the inherent limitation of static weights that fail to differentiate sample difficulty. Furthermore, the exponential smoothing property of EALoss, achieved through the integration of historical IoU values, effectively suppresses gradient oscillations induced by overlapping objects in dense traffic scenarios, thereby improving training stability. The specific formulation for the weighting factor in EALoss is defined as shown in Eqs (10)–(12).


$$d=decay\times \left(1-e^{-\frac{x}{\tau }}\right)\;$$ (10)

Here, *x* represents the current training iteration, τ denotes the time constant used to regulate the smoothing rate of EALoss, and the decay parameter governs the attenuation degree throughout the entire process.


$${modulating}_{weight}=a_{1}\cdot b_{1}+a_{2}\cdot b_{2}+a_{3}\cdot b_{3}\;$$ (11)

$$\left\{ \begin{array}{c} a_{1}=1.0\\ a_{2}=\exp\left(1.0-autoiou\right)\\ a_{3}=\exp\left(-(true-1.0)\right) \end{array}\right.\;$$ (12)

Eqs (11) and (12) define a piecewise weighting function that categorizes samples into three groups—low, intermediate, and high matching degrees—based on the relative position of the ground truth IoU with respect to the dynamic threshold, $auto_{i}ou\;$, and assigns distinct weights to each group. The parameters $a_{1}\;$,$a_{2}\;$ and $a_{3}\;$ correspond to the weighting factors for negative samples, borderline samples, and positive samples, respectively. The definitions of the Boolean variables $b_{1}\;$,$b_{2}\;$ and $b_{3}\;$ are presented in Eq (13).


$$\left\{ \begin{array}{c} b_{1}=\left(true\leq \;{auto}_{iou}-0.1\right)\\ b_{2}=\left({auto}_{iou}-0.1<\;true<\;{auto}_{iou}\right)\\ b_{3}=\left(true\geq \;{auto}_{iou}\right) \end{array}\right.\;$$ (13)

Here, $b_{1}\;$, $b_{2}\;$ and $b_{3}\;$ correspond respectively to samples exhibiting low, moderate, and high levels of agreement between the target and the prediction.

#### Focaler-DIoU function.

In mixed traffic flow scenarios, the original Complete Intersection over Union(CIOU) function exhibits limitations including insufficient sensitivity to small object detection, poor gradient stability, and weak adaptability to complex environments. To address these issues, we incorporate Focaler-IoU [38] and Distance IoU (DIoU) [39], which leverage the characteristics of Focal Loss and DIoU, respectively. The specific computation of IoU is detailed in Eq (14).


$$IoU=\frac{{Area}_{intersection}}{{Area}_{union}}\;$$ (14)

The detailed computation of Focaler-DIoU is presented in Eqs (15)–(18).


$$Focaler\text{-}IoU=\frac{\left(IoU-d\right)}{\left(u-d\right)}\;$$ (15)

$$\rho^{2}=\left(\frac{{\left({b2}_{center}-{b1}_{center}\right)}^{2}}{4}\right)\;$$ (16)

$$c^{2}={𝑐}_{w}^{2}+{𝑐}_{h}^{2}\;$$ (17)

$$Focaler\text{-}DIoU=Focaler\text{-}IoU-\left(\frac{\rho^{2}}{c^{2}}\right)\;$$ (18)

Where *d* denotes the lower bound of Focaler-IoU and u represents its upper bound, both serving to regulate the IoU values. $b1_{center}\;$ and $b2_{center}\;$ correspond to the center coordinates of the predicted and ground truth bounding boxes, respectively; $\rho^{2}\;$ is the squared Euclidean distance between these centers, employed to assess the influence of box localization. $c_{w}\;$ and $c_{h}\;$ signify the width and height of the smallest enclosing box, while $c^{2}\;$ denotes the squared diagonal length of this enclosing box, used for normalizing spatial distance information. The proposed Focaler-DIoU function integrates the focal modulation mechanism with the characteristics of Distance IoU, effectively addressing core challenges in multi-object detection: (1) by introducing dynamic weighting coefficients, it substantially enhances the optimization emphasis on hard samples such as small objects and occlusions, thereby mitigating the imbalance issues caused by fixed weighting in CIOU; (2) through the incorporation of DIoU’s center point distance penalty term, it refines bounding box localization accuracy, enabling more precise spatial topology modeling among targets, particularly in dense mixed traffic flow environments; (3) leveraging the focal modulation mechanism to suppress the gradient dominance of easy samples, it significantly reduces training fluctuations induced by fixed penalty weights in CIOU. Furthermore, this approach dynamically balances the optimization demands across multi-scale targets, improving multi-scale adaptability while maintaining computational efficiency.

## Experiments

### Datasets and implementations

#### Datasets and metrics.

In this paper, we conduct independent training and validation on three publicly available benchmarks: VisDrone2019, UA-DETRAC-G2, and HazyDet. The VisDrone2019 UAV-based remote sensing dataset [40] is utilized following the official partition protocol, with a training, validation, and testing split of approximately 6:1:3. This dataset comprises 10,209 images captured across 14 distinct urban centers, encompassing a broad spectrum of object scales ranging from micro to large instances. It covers diverse urban and rural environments under varying weather and illumination conditions, thereby providing aerial imagery with significant contextual diversity. The UA-DETRAC-G2 traffic surveillance dataset [41] contains over 140,000 frames of RGB imagery, partitioned into training and validation sets at a ratio of approximately 8:2. The images, with a resolution of 960×540  pixels, include four object categories, comprising 8,250 unique vehicles and 1.21 million bounding box annotations. The HazyDet remote sensing dataset [42] consists of 11,000 synthetic images, strictly divided into training, validation, and testing sets with an 8:1:2 ratio. It encompasses three object categories and contains a total of 365,000 object instances. For the generalization experiments, we elect to utilize the original image sets to more authentically reflect the challenges of small object detection in natural environments and to simulate complex target scenarios under diverse weather conditions. This strategy enhances the generalizability and practical relevance of our experiments, overcoming the limitations associated with focusing exclusively on haze effects.

To validate the performance of the proposed method, we employ precision, recall, mean average precision (mAP), and floating point operations (FLOPs) as evaluation metrics. Precision refers to the proportion of correctly classified positive samples among all detected positive samples, whereas recall denotes the proportion of detected positive samples relative to all ground truth positive samples. The formal definitions of precision and recall are presented in Eqs (19) and (20), respectively.


$$Precision=\frac{TP}{TP+FP}\;$$ (19)

$$Recall=\frac{TP}{TP+FN}\;$$ (20)

Here, TP (True Positives) denotes the number of positive samples correctly detected by the model, FP (False Positives) represents the number of negative samples incorrectly classified as positive, and FN (False Negatives) corresponds to the number of positive samples that the model failed to detect. The computation of mAP is detailed in Eqs (21) and (22).


$$AP=\int_{0}^{1}Precision(r)\;dr\;$$ (21)

$$mAP=\frac{1}{N}\sum_{i=1}^{N}{AP}_{i}\;$$ (22)

Here, mAP50 denotes the mean average precision calculated at an Intersection over Union (IoU) threshold of 0.5. Meanwhile, mAP50:95 represents the mean average precision averaged over IoU thresholds ranging from 0.5 to 0.95 with a step size of 0.05.

#### Implementation details.

In this paper, the complete experimental process of the model was conducted on the High-Performance Computing Platform of Qinghai University. The server cluster comprises multiple Lenovo ThinkSystem SR650 nodes with the following specifications: Intel(R) Xeon(R) Silver 4208 CPU @ 2.10GHz and NVIDIA A100 GPUs (80GB). The computational environment utilizes Python 3.10.13 and PyTorch 2.0.1 with CUDA 11.7 support. All experiments were configured with an initial input resolution of 640 × 640 pixels, optimized through stochastic gradient descent (SGD) with a base learning rate of 0.01 and batch size 16. Mosaic data augmentation was consistently applied throughout the training process, while mixed-precision training was explicitly disabled.

### Comparative experiments

To more effectively demonstrate the comparative improvements, this study contrasts the detection performance of the original model with that of our proposed network across various categories within the VisDrone2019 dataset. The results presented in Table 1 indicate that the proposed network achieves significant enhancements over multiple metrics for targets at different scales.

**Table 1:** Comparison of YOLOv11n and MTF-NET on different classes.

	YOLOv11n				MTF-NET			
Class	P	R	mAP50	mAP50:95	P	R	mAP50	mAP50:95
All	41.7	29.9	27.9	15.6	45.6	34.6	33.0	18.5
Pedestrian	46.2	22.6	24.1	9.50	51.5	27.2	29.6	12.0
People	49.0	8.93	12.7	4.45	51.0	15.4	18.8	6.81
Bicycle	20.3	11.4	7.4	12.7	30.5	12.9	11.9	4.92
Car	62.2	68.8	67.6	41.6	67.5	71.8	72.2	45.1
Van	35.9	34.5	31.4	20.1	40.0	42.4	37.5	24.0
Truck	40.4	34.8	30.9	18.8	42.7	40.6	37.0	22.7
Tricycle	22.9	22.8	12.7	6.32	25.4	28.3	17.9	9.40
Awning-tricycle	38.2	17.2	16.0	8.62	37.1	21.4	19.6	11.1
Bus	64.2	47.5	50.8	34.0	66.7	50.1	54.9	36.3
Motor	37.9	30.1	25.4	10.0	43.3	36.0	30.9	12.8

Table notes: Comparison metrics include Precision (P), Recall (R), mAP at IoU 0.5 (mAP50) and mAP averaged over IoU thresholds 0.5 to 0.95 (mAP50:95) for each class.

**Fig 7:**
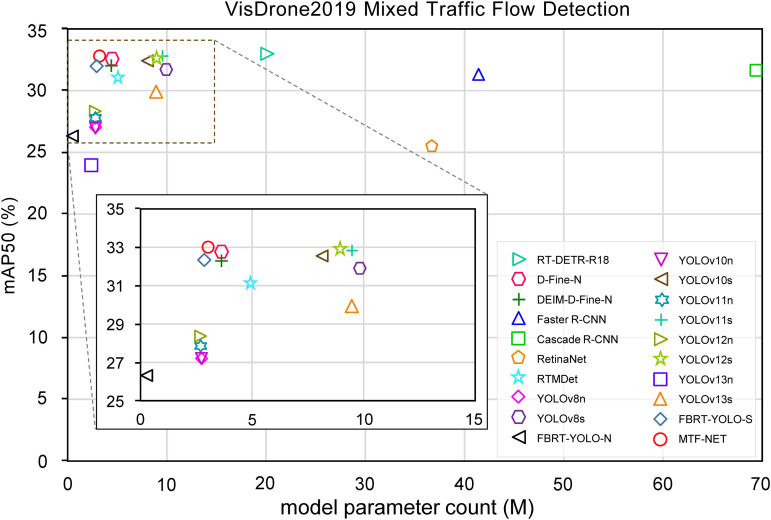
Comparative Analysis of Various Detectors on the VisDrone2019 Dataset.

This paper presents a comprehensive evaluation of model performance on the VisDrone2019 dataset, with the experimental results illustrated in Table 2 and Fig 7. To ensure a fair comparison, all models were trained from scratch, deliberately avoiding the use of pretrained weight initializations. The findings reveal that MTF-NET achieves an mAP50 of 33.0%, thereby demonstrating the best trade-off between detection performance and parameter complexity when compared to both single-stage and two-stage detectors.

**Table 2:** Comparative Analysis of Different Algorithms on the VisDrone2019 Test Set.

Methods	Params/M	GFLOPs	mAP50	mAP50:95
Faster R-CNN	41.39	208.0	31.3	18.4
Cascade R-CNN	69.29	210.0	31.8	18.7
RetinaNet	36.52	210.0	25.6	16.4
RTMDet	4.88	8.0	31.2	18.4
RTDETR-R18 [43]	20.0	60.0	33.2	18.5
D-Fine-N [44]	3.73	7.1	32.8	18.3
DEIM-D-Fine-N [45]	3.73	7.1	32.2	17.7
YOLOv8n	2.69	6.8	27.3	15.3
YOLOv8s	9.83	23.4	32.0	18.4
YOLOv10n	2.70	8.2	27.3	15.4
YOLOv10s	8.04	24.5	32.6	18.8
YOLOv11n	2.58	6.3	27.9	15.6
YOLOv11s	9.42	21.3	32.9	18.9
YOLOv12n	2.56	6.3	28.3	15.4
YOLOv12s	9.23	21.2	33.0	17.6
YOLOv13n	2.45	6.2	24.4	13.3
YOLOv13s	9.00	20.1	29.7	16.7
FBRT-YOLO-N [46]	0.80	6.7	26.5	14.8
FBRT-YOLO-S [46]	2.90	22.9	32.3	18.3
YOLO-NAS-S	19.0	–	29.2	–
YOLO-NAS-M	51.1	–	32.5	–
MTF-NET	3.05	12.4	33.0	18.5

The proposed MTF-NET algorithm exhibits significant advantages in object detection. Compared with two-stage detectors such as Faster R-CNN and Cascade R-CNN, MTF-NET achieves improvements in mAP50 by 1.7% and 1.2%, respectively, while simultaneously reducing the model parameter size and computational complexity by 92.63% and 95.60%. In terms of the speed-accuracy trade-off, MTF-NET outperforms the best-performing single-stage detectors; maintaining a real-time detection speed of 64.3 FPS, it surpasses the mAP50 scores of lightweight variants YOLOv8n, YOLOv10n, YOLOv11n, YOLOv12n, YOLOv13n, and FBRT-YOLO-N by 5.7%, 5.7%, 5.1%, 5.0%, 8.6%, and 6.5%, respectively. Moreover, when compared with RetinaNet and medium-sized models such as YOLOv8s, YOLOv10s, YOLOv11s, and YOLOv13s, MTF-NET demonstrates mAP50 gains of 7.4%, 1.0%, 0.4%, 0.1%, and 3.3%, respectively, while reducing the corresponding parameter size by 91.65%, 68.97%, 62.06%, 67.62%, and 66.11% and the computational complexity by 94.10%, 47.01%, 49.39%, 41.78%, and 38.31%. Although MTF-NET achieves comparable mAP50 detection accuracy to YOLOv12s, it outperforms YOLOv12s by 0.9% in mAP50:95, with a considerably smaller number of parameters and lower computational cost. Furthermore, when compared with the latest DETR models, MTF-NET attains similar detection accuracy to RTDETR-R18, D-Fine-N, and DEIM-D-Fine-N, while utilizing only 15.3%, 81.8%, and 81.8% of their respective parameter sizes. These experimental results demonstrate that the proposed architecture successfully maintains high-precision detection capabilities while ensuring model lightweightness.

#### Comparative experimental results and analysis of the hierarchical implicit-explicit pyramid.

In the multi-target detection task for mixed traffic flow, the model must not only maintain high detection accuracy for large-scale targets but also effectively enhance the performance for small-scale targets. The detection results of the baseline model baseline+A demonstrate that it comprises 2.42M parameters, executes 6.5 GFLOPs, achieves a precision of 41.4%, and attains an mAP50 of 29.0%. To boost small-target detection, we introduced a conventional P2 detection layer and a self-designed feature pyramid on top of baseline+A, with the detailed comparisons presented in Table 3.

**Table 3:** Comparative experiments evaluating various approaches for small-target enhancement.

Methods	P	mAP50	Params/M	GFLOPs
Baseline+A	41.4	29.0	2.42	6.5
Baseline+A + P2	41.7	30.2	2.52	10.7
Baseline+A+HIEP	44.6	30.9	2.90	12.4

Table notes: A represents the improvements derived from CFF/CAF modifications.

After integrating the P2 detection layer, the model’s parameter count increased to 2.52M and the computational load surged significantly to 10.7 GFLOPs. Consequently, the precision improved to 41.7% and mAP50 reached 30.2%. Compared to baseline+A, the P2 layer yielded a 1.2% enhancement in mAP50 while incurring an approximately 65% increase in computational cost. This indicates that, although the conventional P2 layer can partially boost small-target detection performance, it does so at the expense of substantial computational overhead. Furthermore, upon adopting our proposed Hierarchical Implicit-Explicit Pyramid (HIEP), the model achieved a parameter count of 2.9M and a computational burden of 12.4 GFLOPs, with precision rising to 44.6% and mAP50 attaining 30.9%. Relative to baseline+A augmented with the P2 detection layer, the HIEP configuration incurred a roughly 15% increase in parameters and a 16% rise in computational cost, yet realized approximately a 7% improvement in precision and an mAP50 increment of 1.9%. This performance enhancement notably surpasses that provided by simply integrating the P2 detection layer, thereby demonstrating HIEP’s superior capability in capturing and representing features of small targets. In summary, although the introduction of HIEP leads to a marked increase in computational cost, this overhead is justified and cost-effective in light of the substantial boost in performance. Specifically, the mAP50 increased from 29% to 30.9% and precision improved from 41.4% to 44.6%, reflecting significant gains in detection performance. The increments in parameters and computational resources remain within acceptable limits for multi-target detection tasks, indicating that the proposed structure achieves an effective balance between performance and computational demand. Moreover, compared to the traditional P2 detection layer, HIEP, through its layered attention fusion mechanism, more effectively enhances semantic representation and localization accuracy for small targets, thereby evidencing its superiority and practical value.

#### Comparative analysis of the efficacy of different loss functions.

In this study, we compared our proposed EALoss with other conventional loss functions applied individually to the model, based on their performance using the mAP50 and mAP50:95 metrics. The comparison results, detailed in Table 4, demonstrate that EALoss consistently outperforms the alternatives on both metrics, thereby validating the effectiveness of our newly introduced loss function in mixed traffic scenarios.

**Table 4:** Comparative Analysis of the Effectiveness of Different Loss Functions.

LOSS Function	mAP50	mAP50:95
BCELoss	27.9	15.6
FocalLoss	27.5	15.4
EALoss	28.3	16.3

In addition, this study evaluates the performance of various IoU functions when applied individually to the model, using mAP50 and mAP50:95 as the evaluation metrics. The detailed comparison results are presented in Table 5. Notably, when utilized independently, the Focaler-DIoU function achieves the best performance, thereby substantiating its efficacy in enhancing the model’s performance in complex mixed traffic scenarios.

**Table 5:** Comparative Analysis of Different IoU Functions.

IoU Function	mAP50	mAP50:95
CIoU	27.9	15.6
DIoU	27.4	15.4
GIoU	27.3	15.1
SIoU	28.0	15.6
EIoU	27.6	15.5
Focaler-DIoU	28.2	15.8

### Ablation experiments

The detailed results of this ablation experiment are presented in Table 6. Replacing the original C3K2 modules with CCF and CTF leads to a 1.1% increase in mAP50, accompanied by a 3.17% rise in GFLOPs and a 6.2% reduction in parameter count, indicating a substantial enhancement in the model’s ability to capture contextual information. The introduction of the HIEP module results in a 1.9% improvement in mAP50 on the test set by reinforcing cross-scale representation of small objects while preserving the original feature pyramid structure, thereby validating the proposed dual-path calibration mechanism and effectively alleviating information attenuation within the feature pyramid. Substituting the SPPF module with the MKDFN module yields a 1.1% gain in mAP50, with a marginal increase of 0.15M parameters and unchanged computational cost, demonstrating that the dynamic receptive field realized by this module better captures multi-scale object features and adapts to detection demands for objects of varying sizes. Finally, the incorporation of a one-to-one detection head, forming a dynamic dual-head configuration together with the original one-to-many detection head, improves mAP50 by 0.5% without introducing additional computational or parametric overhead. Replacing the original loss and IoU functions with EALoss and Focaler-DIoU further elevates mAP50 by 0.5% without extra computational or parameter burden, enhancing the model’s flexibility in addressing sample imbalance through adaptively adjusting the learning strategy to tackle challenges posed by diverse samples.

**Table 6:** Ablation Study Results on VisDrone2019 Dataset with Various Components and Losses.

CCF/CAF	HIEP	MKDFN	DDDH	EALOSS	FDIoU	P	R	mAP50	GFLOPs	Param/M
						41.7	29.9	27.9	**6.3**	2.58
✓						41.4	30.9	29.0	6.5	**2.42**
	✓					44.1	33.2	31.3	12.3	3.06
✓	✓					44.6	32.3	30.9	12.4	2.90
		✓				40.0	30.4	28.3	**6.3**	2.73
✓	✓	✓				**45.6**	33.6	32.0	12.4	3.05
			✓			40.7	30.3	28.2	**6.3**	2.58
✓		✓	✓			42.8	30.9	29.8	6.5	2.57
✓	✓	✓	✓			45.5	34.0	32.5	12.4	3.05
				✓	✓	40.7	30.7	28.4	6.3	2.58
✓	✓	✓	✓	✓		45.3	34.2	32.7	12.4	3.05
✓	✓	✓	✓	✓	✓	**45.6**	**34.6**	**33.0**	12.4	3.05

Table notes: CCF/CAF, HIEP, MKDFN, DDDH denote different model components; EALOSS and FDIoU indicate loss improvements; P, R, mAP50 represent precision, recall, and mean average precision at IoU 0.5; GFLOPs and Param/M are computational cost and model size.

When these improvements are integrated into the original architecture, the enhanced model achieves a 5.1% increase in mAP50 relative to the baseline, accompanied by improvements of 3.9% in precision and 4.7% in recall. Despite the increased parameter count and computational complexity of the proposed MTF-NET compared to the baseline model, the overhead remains lightweight, with only a 0.47M increase in parameters and an additional 6.3 GFLOPs in computation. These results indicate that the enhanced model maintains superior performance while still meeting the requirements of real-time detection.

### Visualization effects

**Fig 8:**
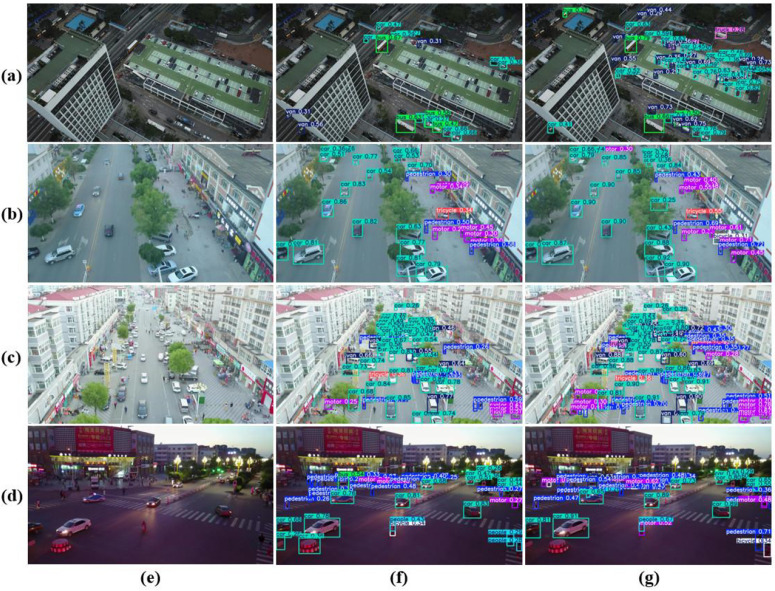
Comparison of detection results between YOLOv11n and MTF-NET under different scenarios. Rows **(a)**, **(b)**, **(c)**, and **(d)** correspond to aerial low-light scenes, occlusion scenes, densely populated object scenes, and nighttime scenes, respectively. Columns **(e)**, **(f)**, and **(g)** represent the original images, detection results from YOLOv11n, and detection results from MTF-NET, respectively.

**Fig 9:**
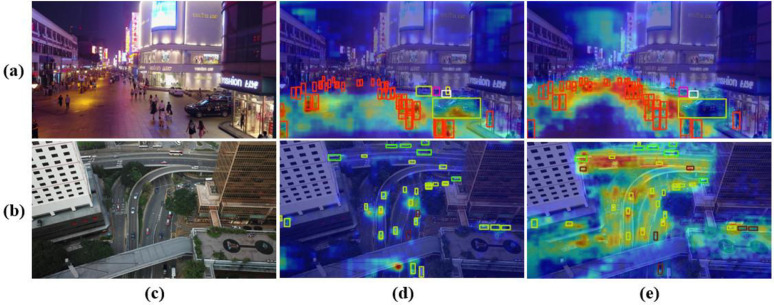
Heatmap visualizations of YOLOv11n and MTF-NET under different viewpoints. Rows **(a)** and **(b)** correspond to nighttime scenes and drone-based aerial small-object scenes, respectively. Columns **(c)**, **(d)**, and **(e)** represent the original images, heatmaps generated by YOLOv11n, and heatmaps generated by MTF-NET, respectively.

In this study, visualization results were employed to directly demonstrate the performance improvements achieved by the refined model. Fig 8 presents a comparative visualization of detection outcomes, where the second column displays the detection results from YOLOv11n and the third column shows those from the enhanced MTF-NET. The columns correspond respectively to high-altitude aerial views, occlusion, dense target scenarios, and low-light conditions. Our method significantly reduces both missed detections and false positives, thereby validating the effectiveness of the proposed multi-scale feature extraction module, hierarchical feature modeling strategy, and cross-level feature fusion architecture. Particularly for detection tasks under complex occlusion and extreme viewpoint conditions, the dynamic feature enhancement mechanism effectively suppresses the interference caused by target appearance degradation, further underscoring the robustness of the approach. Fig 9 further illustrates a side-by-side comparison of feature heatmap visualizations between the baseline and the improved model in the second and third columns, respectively. The results indicate a substantial enhancement in the refined model’s feature focusing capability, a performance gain attributed to the dynamic global feature interactions realized through the self-attention mechanism within the CAF module. The collaboration between the self-attention mechanism and the multi-head architecture effectively overcomes the inherent limitations of local receptive fields in traditional convolutional neural networks, thereby significantly enhancing the model’s ability to represent complex target features.

### Generalization Verification

**Fig 10:**
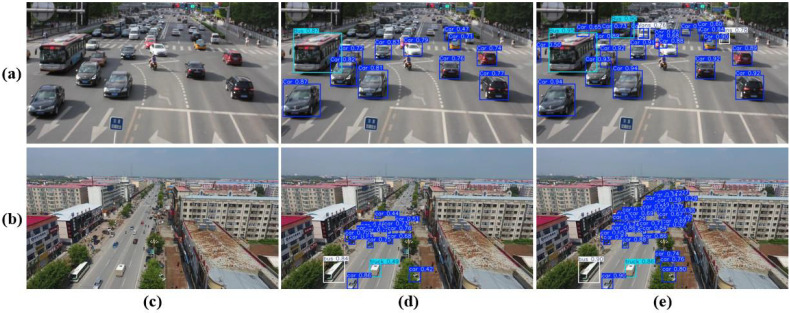
A comparison of detection performance between YOLOv11n and MTF-NET on the UA-DETRAC-G2 dataset (a) and the original HazyDet dataset (b). Row **(a)** presents sampled visualized detection outcomes for the UA-DETRAC-G2 dataset, while row **(b)** illustrates corresponding results for the HazyDet dataset. In both cases, column **(c)** displays the original images, column **(d)** presents the detection results from YOLOv11n, and column **(e)** shows the outcomes achieved by MTF-NET.

This paper validates the generalization capability of the proposed algorithm through comprehensive experiments conducted on the UA-DETRAC-G2 and HazyDet original datasets. The comparative performance between the baseline and the improved models on these datasets is illustrated in Fig 10. The UA-DETRAC-G2 dataset predominantly comprises medium and small-sized targets, whereas the HazyDet dataset mainly consists of small and ultra-small targets.

Experimental results are summarized in Tables 7 and 8: on the UA-DETRAC-G2 dataset, the improved algorithm achieves increases of 1.4%, 6.0%, 4.2%, and 3.2% in Precision, Recall, mAP50, and mAP50:95, respectively, with notable gains observed across all category-specific mAP metrics, thereby effectively validating the algorithm’s applicability. The generalization experiments on the HazyDet test set demonstrate improvements of 9.1%, 12.6%, 13.4%, and 12.6% in overall Precision, Recall, mAP50, and mAP50:95, respectively, with significant enhancements also evident in single-category metrics. These findings substantiate the efficacy of the proposed improved algorithm in mixed traffic flow scenarios dominated by small and ultra-small targets, thereby comprehensively confirming its generalization capability.

**Table 7:** Generalization experiments on the UA-DETRAC-G2 dataset.

Method and Category		Precision	Recall	mAP50	mAP50:95
YOLOv11n	**All**	**60.9**	**44.1**	**50.2**	**35.9**
	Car	75.7	62.1	70.7	49.1
	Bus	80.7	65.2	72.6	52.0
	Vans	61.6	44.7	48.1	36.2
	Others Vehicles	25.8	4.35	9.18	6.21
MTF-NET	**All**	**62.3**	**50.1**	**54.4**	**39.1**
	Car	76.5	67.4	73.9	52.4
	Bus	74.7	71.0	74.2	52.2
	Vans	51.5	53.5	51.8	39.5
	Others Vehicles	46.5	8.48	17.8	12.5

**Table 8:** Generalization experiments conducted on the test set of the original HazyDet dataset.

Method and Category		Precision	Recall	mAP50	mAP50:95
YOLOv11n	**All**	**75.3**	**57.3**	**63.8**	**43.0**
	Car	86.2	76.8	83.1	54.0
	Truck	61.8	29.7	36.4	22.8
	Bus	78.0	65.5	71.9	52.2
MTF-NET	**All**	**84.4**	**69.9**	**77.2**	**55.6**
	Car	91.6	83.0	89.7	64.3
	Truck	74.7	48.9	57.4	38.1
	Bus	86.8	77.9	84.6	64.5

## Conclusion

In this paper, we propose the MTF-NET framework to effectively address the series of multi-scale object detection challenges inherent in mixed traffic scenarios. By integrating the global and local feature representations through the ContextAttentionFormer and ConvFuseFormer architectures, and enhancing the multi-scale feature expressive capabilities with a hierarchical implicit-explicit pyramid coupled with a multi-kernel dilation fusion network, our approach provides robust detection mechanisms. The designed EALoss and Focaler-DIoU loss functions offer an effective gradient-balanced self-adaptive optimization strategy. Experimental results demonstrate that MTF-NET achieves a mAP50 of 33.0% on the VisDrone2019 test set, outperforming YOLOv11n by 5.1% while incurring only an additional 0.47M parameters, thereby exhibiting an excellent balance between performance and efficiency. The proposed method demonstrates strong generalization and robustness, making it suitable for real-time object detection tasks in diverse and complex traffic environments, and it holds significant promise for applications in intelligent transportation surveillance, UAV inspection, and urban traffic management.

## Supporting information

S1 Fig Details of the VisDrone2019 Dataset. The figure depicts the category distribution and associated annotation information of the VisDrone2019 dataset.
(PNG)

S2 Fig Details of the UA-DETRAC-G2 Dataset. The figure depicts the category distribution and associated annotation information of the UA-DETRAC-G2 dataset. The UA-DETRAC-G2 dataset constitutes a publicly accessible subset of the UA-DETRAC dataset.
(PNG)

S3 Fig Details of the HazyDet Dataset. The figure depicts the category distribution and associated annotation information of the HazyDet dataset.
(PNG)

S1 File Experimental data. This file contains the data of the experiment, datails of which are described below. **Table 1.** Comparison of Different Classes in the VisDrone2019 Test Set. **Table 2.** Comparative Analysis of Different Algorithms Based on the VisDrone2019 Test Set. **Table 3.** Comparative Experiments on Different Small-Object Enhancement Methods. **Table 4.** Comparative Analysis of the Effects of Different Loss Functions. **Table 5.** Comparative Analysis of the Performance of Various IoU Functions. **Table 6.** Ablation Experimental Results on the Test Set of the VisDrone2019 Dataset. **Table 7.** Generalization Experiments on the UA-DETRAC-G2 Dataset. **Table 8.** Generalization Experiments on the Test Set of the Original HazyDet Dataset.
(ZIP)
