# Supplementary figures and images for: MTF-NET: A mixed traffic flow multi-target detection network based on full-field perception and adaptive optimization
Source: PLoS One. 2026 Mar 16;21(3):e0344151. doi: 10.1371/journal.pone.0344151 (PMC12991249; doi:10.1371/journal.pone.0344151)

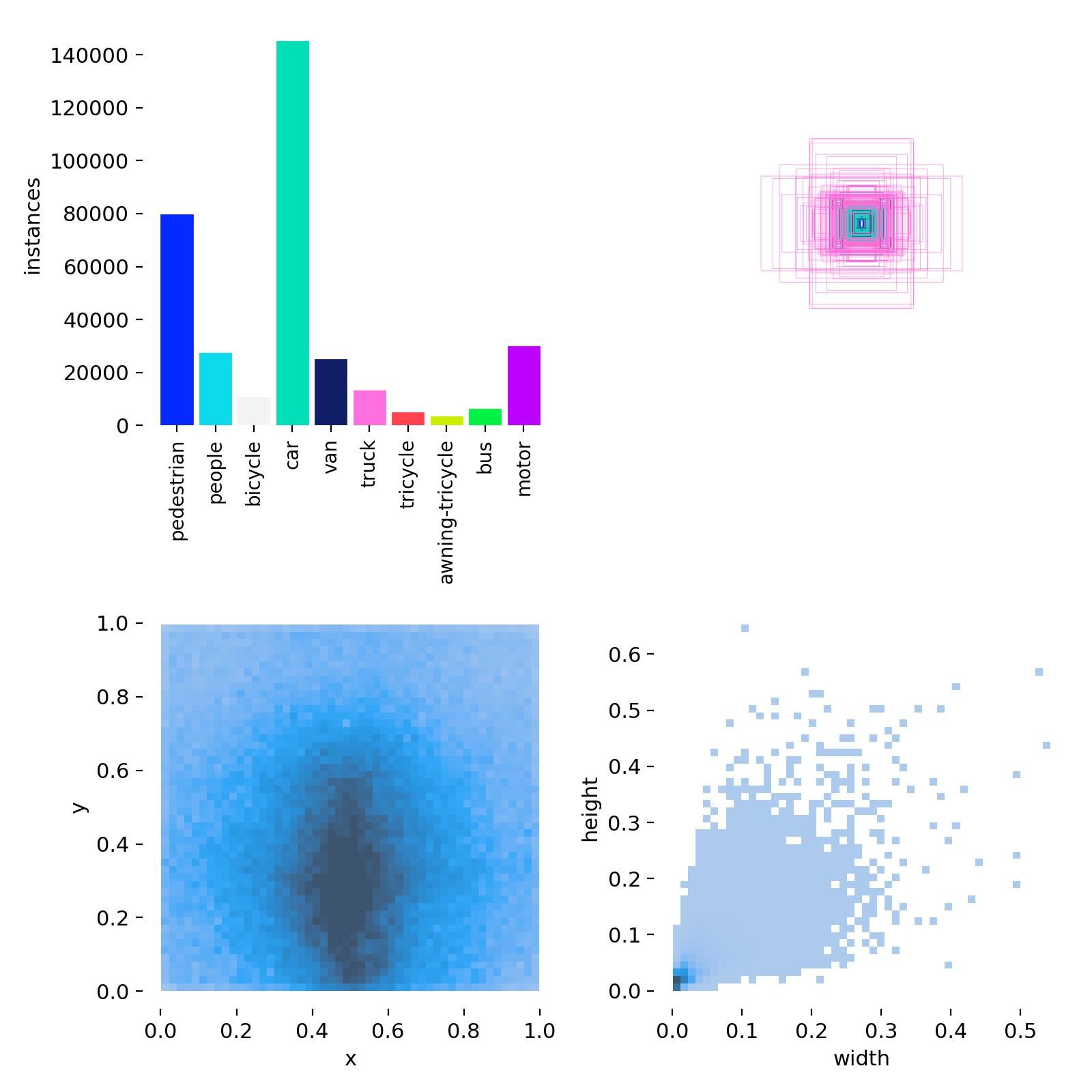

Supplement: S1 Fig — The figure depicts the category distribution and associated annotation information of the VisDrone2019 dataset. (PNG) [file pone.0344151.s001.png]

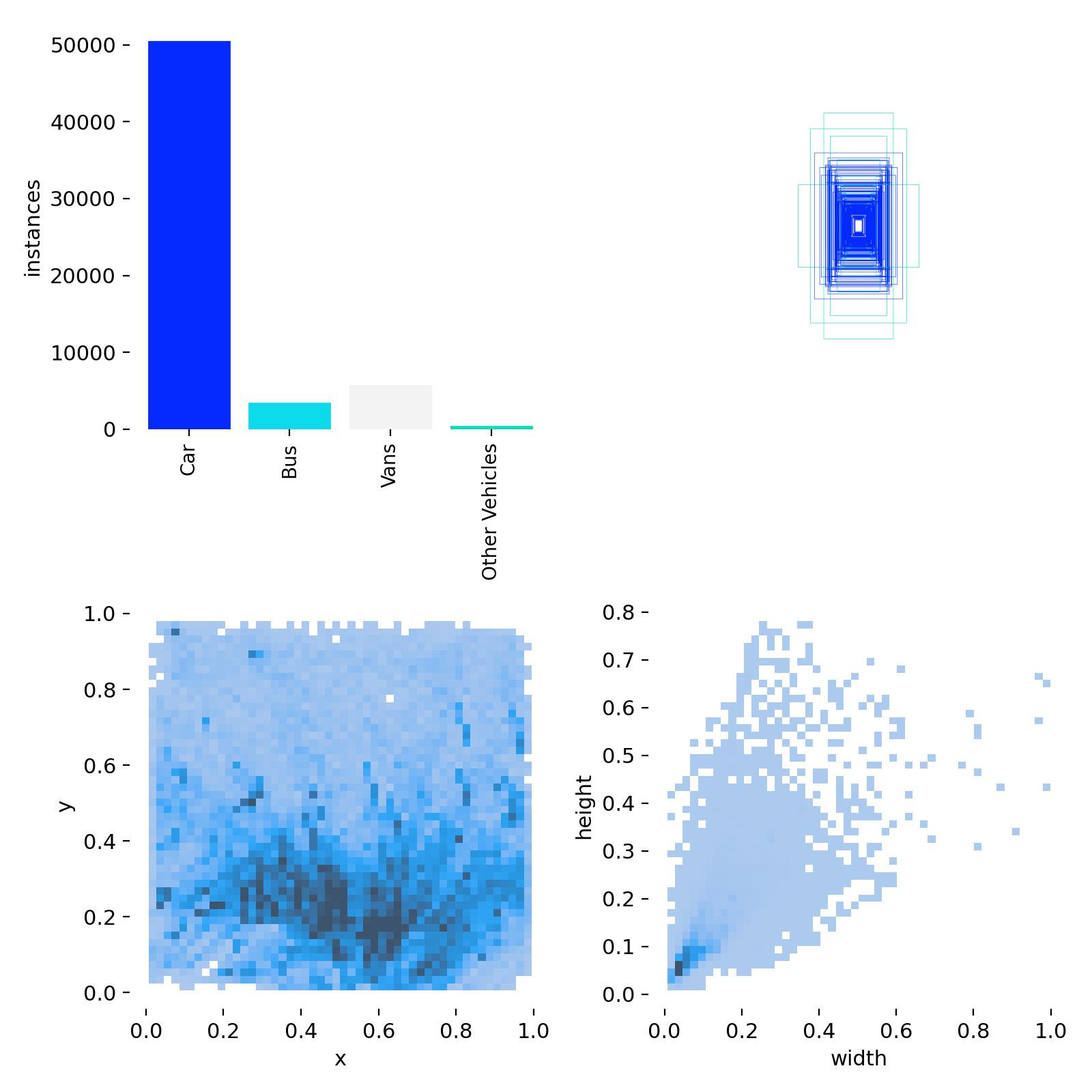

Supplement: S2 Fig — The figure depicts the category distribution and associated annotation information of the UA-DETRAC-G2 dataset. The UA-DETRAC-G2 dataset constitutes a publicly accessible subset of the UA-DETRAC dataset. (PNG) [file pone.0344151.s002.png]

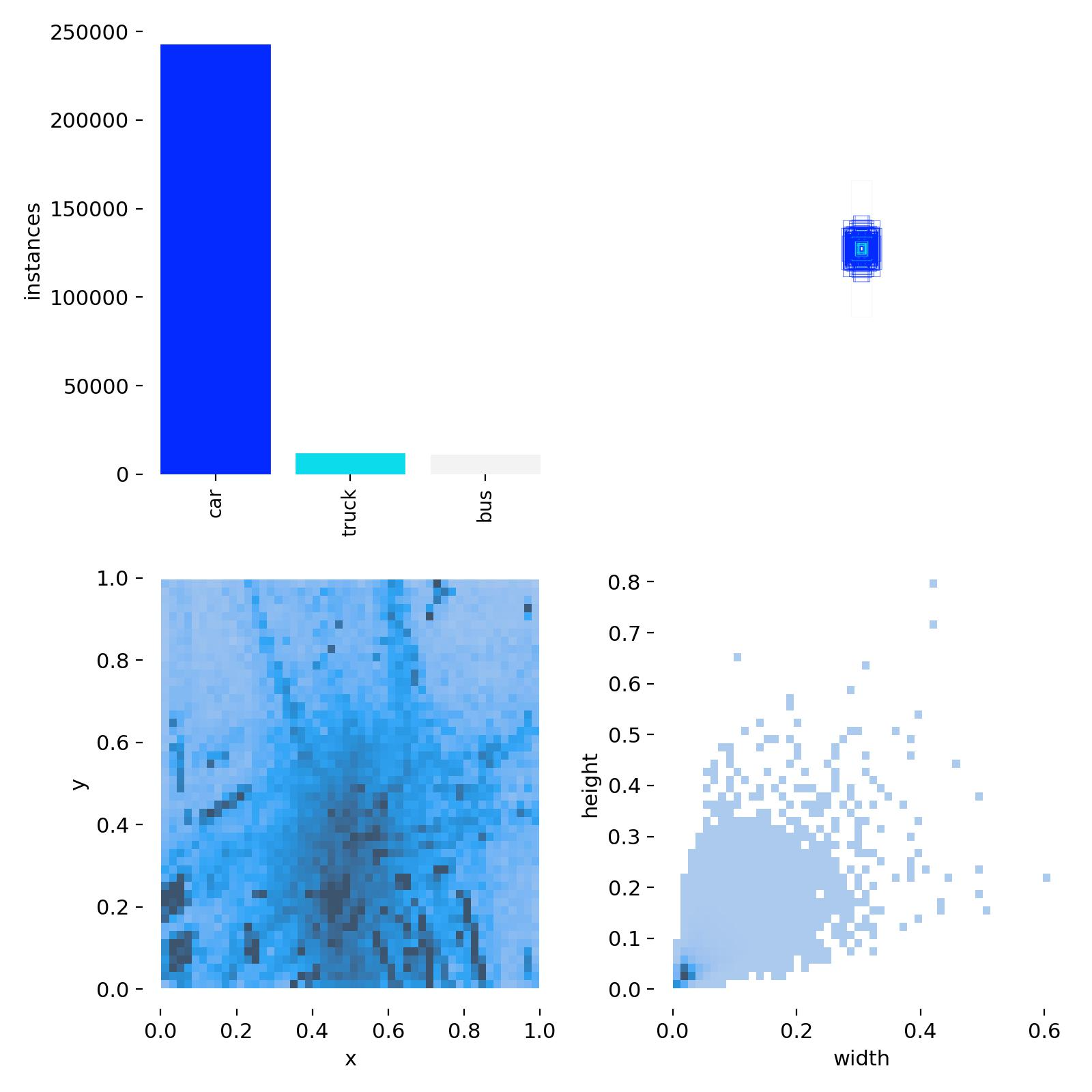

Supplement: S3 Fig — The figure depicts the category distribution and associated annotation information of the HazyDet dataset. (PNG) [file pone.0344151.s003.png]
