# Supplementary material for: MTF-NET: A mixed traffic flow multi-target detection network based on full-field perception and adaptive optimization
Source: PLoS One. 2026 Mar 16;21(3):e0344151. doi: 10.1371/journal.pone.0344151 (PMC12991249; doi:10.1371/journal.pone.0344151)
Supplement: S1 File — This file contains the data of the experiment, datails of which are described below. Table 1. Comparison of Different Classes in the VisDrone2019 Test Set. Table 2. Comparative Analysis of Different Algorithms Based on the VisDrone2019 Test Set. Table 3. Comparative Experiments on Different Small-Object Enhancement Methods. Table 4. Comparative Analysis of the Effects of Different Loss Functions. Table 5. Comparative Analysis of the Performance of Various IoU Functions. Table 6. Ablation Experimental Results on the Test Set of the VisDrone2019 Dataset. Table 7. Generalization Experiments on the UA-DETRAC-G2 Dataset. Table 8. Generalization Experiments on the Test Set of the Original HazyDet Dataset. (ZIP) [file pone.0344151.s004.zip › S1_File/Table7.docx]

**TABLE 7** Generalization Experiments on the UA-DETRAC-G2 Dataset.

| **Method** | **Category** | **Precision** | **Recall** | **mAP50** | **mAP50:95** |
| --- | --- | --- | --- | --- | --- |
| YOLOv11n | **All** | **60.9** | **44.1** | **50.2** | **35.9** |
|  | Car | 75.7 | 62.1 | 70.7 | 49.1 |
|  | Bus | 80.7 | 65.2 | 72.6 | 52.0 |
|  | Vans | 61.6 | 44.7 | 48.1 | 36.2 |
|  | Others Vehicles | 25.8 | 4.35 | 9.18 | 6.21 |
| MTF-NET | **All** | **62.3** | **50.1** | **54.4** | **39.1** |
|  | Car | 76.5 | 67.4 | 73.9 | 52.4 |
|  | Bus | 74.7 | 71.0 | 74.2 | 52.2 |
|  | Vans | 51.5 | 53.5 | 51.8 | 39.5 |
|  | Others Vehicles | 46.5 | 8.48 | 17.8 | 12.5 |
